# General anesthesia vs. non-general anesthesia for elderly stroke patients treated with mechanical thrombectomy

**DOI:** 10.3389/fneur.2026.1753649

**Published:** 2026-03-12

**Authors:** Wanying Shan, Mengyi Xu, Xiuqun Gong, Yi Xie, Xiaohao Zhang, E. Yan, Hongmei Zhu, Yishan Lei, Liang Xu

**Affiliations:** 1 Department of Neurology, Suzhou Ninth People’s Hospital, Soochow University, Suzhou, Jiangsu, China; 2 Department of Neurology, Nanjing First Hospital, Nanjing Medical University, Nanjing, Jiangsu, China; 3 Department of Neurology, The First Hospital of Anhui University of Science and Technology, Huainan, Anhui, China; 4 Department of Anesthesiology, Suzhou Xiangcheng People’s Hospital, Suzhou, Jiangsu, China; 5 Department of Anesthesiology, The First Affiliated Hospital of Soochow University, Suzhou, Jiangsu, China

**Keywords:** anesthesia choice, elderly patients, function outcome, stroke, thrombectomy

## Abstract

**Objective:**

This study aimed to compare the impact of general anesthesia (GA) versus non-GA on functional outcomes in elderly patients undergoing endovascular thrombectomy (EVT) for acute ischemic stroke.

**Methods:**

A total of 707 elderly stroke patients (mean age 74.7 ± 6.8 years; 57.0% male) who received EVT were retrospectively analyzed. Patients were stratified into GA (48.1%) and non-GA (51.9%) groups. The primary outcome was the rate of good functional outcome, defined as a modified Rankin Scale (mRS) score of 0–2 at 90 days. Secondary outcomes included successful reperfusion (mTICI 2b-3), symptomatic intracerebral hemorrhage (sICH), early neurological deterioration, post-stroke pneumonia, mortality, and median mRS score at 90 days.

**Results:**

Baseline characteristics were comparable between the GA and non-GA groups, except for a marginally longer onset to groin puncture time in the GA group (*p* = 0.092). The primary outcome of good functional recovery at 90 days was not significantly different between the GA and non-GA groups (46.8% vs. 44.1%; adjusted odds ratio [aOR] 1.359, 95% confidence interval [CI] 0.913–2.022; *p* = 0.131). No significant differences were observed in successful reperfusion, sICH, early neurological deterioration, mortality, or median mRS scores. However, the GA group had a significantly higher incidence of post-stroke pneumonia compared to the non-GA group (37.9% vs. 27.5%; aOR 1.668, 95% CI 1.197–2.325; *p* = 0.003).

**Conclusion:**

In elderly stroke patients undergoing EVT, the type of anesthesia was not associated with significant differences in 90-day functional outcomes. However, GA was linked to a higher risk of post-stroke pneumonia.

## 1 Introduction

Acute ischemic stroke represents a major cause of mortality and long-term disability worldwide, particularly among the elderly (1, 2). Endovascular thrombectomy (EVT) has become the standard of care for eligible patients with large vessel occlusion (3). However, the optimal choice of anesthesia modality for EVT remains a subject of debate (4, 5). General anesthesia (GA) may offer better procedural conditions by minimizing patient movement, yet it carries potential risks such as hemodynamic instability and delayed intervention (6, 7). Conversely, procedural sedation (PS) and local anesthesia (LA) allows for faster workflow and avoids the cardiovascular effects of GA, but may be associated with patient agitation or compromised airway protection (8).

Previous studies and meta-analyses have yielded conflicting results regarding the impact of anesthesia type on functional outcomes, with some suggesting better outcomes under GA and others favoring PS/LA or showing no difference (5, 9–11). This inconsistency may be partly explained by variations in patient characteristics, procedural protocols, and outcome definitions. Importantly, constituting a growing proportion of stroke cases, elderly patients often present with more comorbidities and may be more vulnerable to anesthesia-related complications (12). We hypothesized that anesthesia choice is linked to differential risks of complications and recovery profiles in this vulnerable population. Nevertheless, the literature specifically examining this age group is lacking.

Hence, we performed a multi-center study to evaluate the association of anesthetic strategies with procedure-related and clinical outcomes in elderly patients undergoing EVT for acute ischemic stroke.

## 2 Methods

### 2.1 Study design and participating centers

This retrospective multicenter cohort study included ischemic stroke patients receiving EVT who were prospectively enrolled in 5 stroke centers in China (Center 1: Suzhou Ninth People’s Hospital, April 2021–March 2025; Center 2: W Nanjing First Hospital, January 2024–December 2024; Center 3: The First Hospital of Anhui University of Science and Technology, September 2023–March 2025; Center 4: Suzhou Xiangcheng People’s Hospital, July 2019–June 2025; Center 5: The First Affiliated Hospital of Soochow University, January 2019–June 2025). The inclusion criteria for this study were as follows: (1) aged greater than or equal to 65 years; (2) patients with acute ischemic stroke due to proximal large vessel occlusion (internal carotid artery and/or M1-M2 segment of the middle cerebral artery). We further excluded patients whose anesthetic method was not specifically recorded or who had converted from PS/LA to GA. The remaining patients were divided into two groups according to the anesthesia type: the GA group and the non-GA group. The non-GA group comprised patients who received PS or LA only. Patients in the non-GA group were administered a subcutaneous injection of Xylocaine, supplemented when needed with low-dose short-acting analgesic or sedative agents. Patients in GA received analgesics and/or sedatives at higher doses at the discretion of anesthetists. For patients treated with GA, early extubation was targeted whenever feasible. The study complied with the Helsinki Declaration and received approval from the institutional review board at each participating center. Due to the retrospective design, the Ethics Committee granted a waiver for informed consent.

### 2.2 Baseline data collection and assessment criteria

Baseline data were collected prospectively at the time of admission and included: 1. Demographic variables: age and sex; 2. Clinical variables: baseline BP levels, medical history, stroke severity, stroke etiology, and infarct volume. Stroke severity was assessed using National Institutes of Health Stroke Scale (NIHSS). Pre-treatment infarct volume was measured by the Alberta stroke program early computerized tomography score (ASPECT) (13). Stroke subtype was classified according to the criteria of Trial of Org 10,172 in Acute Stroke Treatment (14); 3. Radiographic variables: occlusive site and collateral circulation. Collateral status was assessed using the American Society of Interventional and Therapeutic Neuroradiology/Society of Interventional Radiology grading system, with grade 0–1 indicating poor collateral circulation and grade 2–4 indicating moderate to excellent (15); 4. Treatment characteristics: prior t-PA treatment, time intervals, time of EVT passes, and degree of reperfusion. Successful reperfusion was defined as modified Thrombolysis in Cerebral Infarction score of 2b or 3 (16, 17); 5. Laboratory variables: baseline blood glucose and high-sensitivity C-reactive protein (hs-CRP).

### 2.3 Outcome measures

The primary outcome measure was the favorable outcome, defined as a modified Rankin Scale (mRS) score of 0–2 at 90 days. Secondary outcomes included the rate of successful reperfusion, the distribution of mRS score, early neurological deterioration (END) and post-stroke pneumonia. Safety outcomes included all-cause mortality at 90 days and the rates of symptomatic intracranial hemorrhage (sICH). The sICH was diagnosed within 24 h of EVT treatment using the Heidelberg Bleeding Classification (18, 19). END was defined as an increase of ≥ 4 points in NIHSS score within 24 h after EVT (20, 21). Post-stroke pneumonia was diagnosed in accordance with the modified Centers for Disease Control and Prevention criteria for hospital-acquired pneumonia. The diagnosis, made by trained clinicians based on clinical and laboratory parameters of acute lower respiratory tract infection, was supported by radiographic confirmation (22, 23).

### 2.4 Statistical analysis

Continuous variables were described as the mean (SD) or median (IQR) as appropriate. Categorical variables were described as numbers (percentage). Normality of distributions was assessed using histograms and the Shapiro–Wilk test. Categorical variables were examined using the *χ*^2^ test. Mann–Whitney U test was used for skewed data, while Student’s *t*-test was used for normally distributed data.

The effects of the anesthetic approach (GA vs. non-GA) were estimated by logistic regression analyses. All multivariable analyses were adjusted for potential confounders included demographic characteristics, hypertension, diabetes mellitus, baseline NIHSS score, pre-treatment ASPECTS, poor collateral status, No. of passes, levels of baseline blood glucose and Hs-CRP. Several sensitivity analyses were conducted to test the robustness of our findings. Furthermore, ordinal regression analysis was used to explore the association between anesthetic choice and the distribution of mRS score. Adjusted odds ratios (OR) were reported, along with their *p* values and 95% confidence interval (CI). Statistical significance was defined as a *p* value of <0.05. All analyses were processed using SPSS version 26 (IBM Corp., Armonk, NY).

## 3 Results

### 3.1 Study population and baseline characteristics

A total of 707 elderly stroke patients (mean age 74.7 ± 6.8 years; 57.0% male) who underwent EVT were included in this study. The most prevalent vascular risk factor was hypertension (76.2%), followed by diabetes mellitus (35.2%), current smoking (36.4%), coronary heart disease (17.4%), and hyperlipidemia (10.9%). The median baseline NIHSS score was 14.0, and median pre-treatment ASPECTS was 9.0. According to the TOAST, the most common stroke etiology was large-artery atherosclerosis (48.7%), followed by cardio-embolism (42.3%). The majority of occlusions were located in the middle cerebral artery (60.7%). Regarding to treatment characteristics, 36.5% of patients received tPA treatment before EVT, and the median onset to groin puncture time was 276.5 min.

Of these, 340 patients underwent GA (48.1%) and 367 underwent PS/LA (51.9%). Patients under GA had a slightly longer onset to groin puncture time than those in non-GA group (*p* = 0.092). However, no statistically significant differences observed across other demographic characterstics, clinical, and treatment characteristics (all *p* > 0.05) (see Table 1).

**Table 1:** Baseline characteristics according to the anesthesia choice.

Variables	All patients (*n* = 707)	General anesthesia (*n* = 340)	Non-general anesthesia (*n* = 367)	*p* value
Demographic characteristics				
Age, years	74.7 ± 6.8	75.1 ± 7.0	74.3 ± 6.6	0.137
Male, *n* (%)	403 (57.0)	194 (57.1)	209 (56.9)	0.976
Risk factors, *n* (%)				
Hypertension	539 (76.2)	262 (77.1)	277 (75.1)	0.621
Diabetes mellitus	249 (35.2)	123 (36.2)	126 (34.3)	0.608
Hyperlipidemia	77 (10.9)	42 (12.4)	35 (9.5)	0.230
Current smoking	257 (36.4)	122 (35.9)	135 (36.8)	0.803
Coronary heart disease	123 (17.4)	60 (17.6)	63 (17.2)	0.866
Clinical characteristics				
Systolic blood pressure, mmHg	138.5 ± 21.9	139.1 ± 21.6	137.9 ± 22.2	0.501
Diastolic blood pressure, mmHg	84.3 ± 13.5	84.5 ± 13.7	84.2 ± 13.5	0.767
Baseline NIHSS, score	14.0 (10.0, 18.0)	14.0 (10.0, 18.0)	14.0 (11.0, 18.0)	0.186
Pre-treatment ASPECTS, score	9.0 (8.0, 9.0)	9.0 (8.0, 9.0)	9.0 (8.0, 9.0)	0.487
Stroke etiology, *n* (%)				0.206
Large-artery atherosclerosis	344 (48.7)	169 (49.7)	175 (47.7)	
Cardio-embolism	299 (42.3)	147 (43.2)	152 (41.4)	
Others/unknown	64 (9.1)	24 (7.1)	40 (10.9)	
Poor collateral status, *n* (%)	374 (52.9)	181 (53.2)	193 (52.6)	0.863
Occlusive site, *n* (%)				0.569
Internal carotid artery	278 (39.3)	130 (38.2)	148 (40.3)	
Middle cerebral artery	429 (60.7)	210 (61.8)	219 (59.7)	
Laboratory data				
Baseline blood glucose, mmol/L	7.3 ± 2.4	7.2 ± 2.4	7.3 ± 2.3	0.742
Hs-CRP, mg/L	11.2 (6.4, 23.2)	10.9 (6.6, 22.6)	11.5 (6.0, 23.5)	0.754
Treatment characteristics				
Prior tPA treatment, *n* (%)	258 (36.5)	130 (38.2)	128 (34.9)	0.354
Onset to groin puncture, min (IQR)	276.5 (175.0, 433.0)	285.0 (183.5, 450.5)	261.0 (169.0, 400.0)	0.092
Groin puncture to reperfusion, min (IQR)	60.0 (45.0, 85.0)	60.0 (45.0, 85.0)	60.0 (44.0, 89.0)	0.651
Onset to reperfusion, min (IQR)	345.0 (242.0, 507.0)	345.0 (247.0, 531.0)	347.5 (235.0, 487.0)	0.186
No. of passes (IQR)	1.0 (1.0, 2.0)	1.0 (1.0, 2.0)	1.0 (1.0, 2.0)	0.936

ASPECTS, the Alberta stroke program early computed tomography score; Hs-CRP, Hyper-sensitive C-reactive protein; NIHSS, National institute of health stroke scale; tPA, Tissue-type plasminogen activator.

### 3.2 Primary and secondary outcomes by anesthesia type

During the 90-day follow-up, 321 patients (45.4%) experienced a poor functional outcome. Table 2 presented a comparison of the baseline characteristics stratified by functional outcome. Patients with a poor functional outcome (*n* = 321) were significantly older (75.9 ± 7.1 years vs. 73.4 ± 6.4 years; *p* = 0.001), had a higher prevalence of hypertension (81.6% vs. 71.8%; *p* = 0.002) and diabetes mellitus (45.8% vs. 26.4%; *p* = 0.001), presented with higher baseline NIHSS scores (median, 17.0 vs. 12.0; *p* = 0.001), lower pre-treatment ASPECTS (median, 9.0 vs. 9.0; *p* = 0.001), a higher rate of poor collateral status (62.0% vs. 45.3%; *p* = 0.001), elevated baseline blood glucose (7.9 ± 2.5 mmol/L vs. 6.7 ± 2.1 mmol/L; *p* = 0.001) and Hs-CRP levels (median, 15.3 mg/L vs. 9.2 mg/L; *p* = 0.001), required a higher number of EVT passes (2.0 vs. 1.0; *p* = 0.001), and had lower rates of successful reperfusion (73.8% vs. 92.0%; *p* = 0.001) compared to patients with favorable functional outcome. They also experienced significantly higher incidences of post-stroke pneumonia (36.4% vs. 29.3%; *p* = 0.043), sICH (19.9% vs. 4.4%; *p* = 0.001), and early neurological deterioration (30.2% vs. 8.5%; *p* = 0.001).

**Table 2:** Baseline characteristics stratified by functional outcome.

Variables	Functional outcome at 90 days		*p* value
	Good outcome (*n* = 386)	Poor outcome (*n* = 321)	
Demographic characteristics			
Age, years	73.4 ± 6.4	75.9 ± 7.1	0.001
Male, *n* (%)	229 (59.3)	174 (56.2)	0.171
Risk factors, *n* (%)			
Hypertension	277 (71.8)	262 (81.6)	0.002
Diabetes mellitus	102 (26.4)	147 (45.8)	0.001
Hyperlipidemia	37 (11.5)	40 (10.4)	0.621
Current smoking	109 (34.0)	148 (38.3)	0.227
Coronary heart disease	62 (19.3)	61 (15.8)	0.226
Clinical characteristics			
Systolic blood pressure, mmHg	137.5 ± 21.6	139.7 ± 22.3	0.168
Diastolic blood pressure, mmHg	84.8 ± 13.3	83.7 ± 13.9	0.274
Baseline NIHSS, score	12.0 (8.0, 14.0)	17.0 (14.0, 22.0)	0.001
Pre-treatment ASPECTS, score	9.0 (8.0, 10.0)	9.0 (8.0, 9.0)	0.001
Stroke etiology, *n* (%)			0.102
Large-artery atherosclerosis	182 (47.2)	162 (50.5)	
Cardio-embolism	161 (41.7)	138 (43.0)	
Others/unknown	43 (11.1)	21 (6.5)	
Poor collateral status, *n* (%)	175 (45.3)	199 (62.0)	0.001
Occlusive site, *n* (%)			0.229
Internal carotid artery	144 (37.3)	134 (41.7)	
Middle cerebral artery	242 (62.7)	187 (58.3)	
General anesthesia, *n* (%)	181 (46.9)	159 (49.5)	0.484
Laboratory data			
Baseline blood glucose, mmol/L	6.7 ± 2.1	7.9 ± 2.5	0.001
Hs-CRP, mg/L	9.2 (4.5, 17.2)	15.3 (8.5, 31.7)	0.001
Treatment characteristics			
Prior tPA treatment, *n* (%)	140 (36.3)	118 (36.8)	0.893
Door to groin puncture, min (IQR)	271.0 (163.0, 441.0)	285.0 (193.0, 433.0)	0.145
Onset to groin puncture, min (IQR)	61.0 (45.0, 85.0)	60.0 (44.0, 85.0)	0.725
Groin puncture to reperfusion, min (IQR)	339.0 (230.0, 510.0)	357.5 (253.0, 501.0)	0.715
No. of passes (IQR)	1.0 (1.0, 2.0)	2.0 (1.0, 3.0)	0.001
Successful reperfusion, *n* (%)	355 (92.0)	237 (73.8)	0.001
Post-stroke pneumonia, *n* (%)	113 (29.3)	117 (36.4)	0.043
sICH, *n* (%)	17 (4.4)	64 (19.9)	0.001
Early neurological deterioration, *n* (%)	33 (8.5)	97 (30.2)	0.001

ASPECTS, the Alberta stroke program early computed tomography score; END, Early neurological deterioration; Hs-CRP, Hyper-sensitive C-reactive protein; sICH, Symptomatic intracerebral hemorrhage NIHSS, National institute of health stroke scale; tPA, Tissue-type plasminogen activator.

Table 3 displayed the effect of anesthesia choice in procedure-related and clinical outcomes in elderly stroke patients after EVT. The primary outcome was comparable between the GA and non-GA groups (46.8% vs. 44.1%; aOR 1.359, 95% CI 0.913–2.022; *p* = 0.131).

**Table 3:** Outcomes by anesthesia choice in elderly stroke patients receiving mechanical thrombectomy.

Outcome	General anesthesia	Non-general anesthesia	aOR (95%CI)*	*P* value
Primary outcome				
Good functional outcome at 90 days, No/total No. (%)	159/340 (46.8)	162/367 (44.1)	1.359 (0.913–2.022)	0.131
Secondary outcomes				
Successful reperfusion, No/total No. (%)	295/340 (86.8)	297/367 (80.9)	1.372 (0.886–2.125)	0.157
sICH, No/total No. (%)	44/340 (12.9)	37/367 (10.1)	1.444 (0.835–2.443)	0.471
Early neurological deterioration, No/total No. (%)	63/340 (18.5)	67/367 (18.3)	0.990 (0.658–1.490)	0.963
Post-stroke pneumonia, No/total No. (%)	129/340 (37.9)	101/367 (27.5)	1.668 (1.197–2.325)	0.003
mRS at 90 days, median (IQR)	2.0 (1.0, 5.0)	2.0 (1.0, 5.0)	0.935 (0.721–1.213)	0.612
Mortality at 90 days, No/total No. (%)	63/340 (18.5)	84/367 (21.9)	0.758 (0.483–1.187)	0.226

CI, confidence interval; aOR, adjusted add ratio.

*adjusted for demographic characteristics, hypertension, diabetes mellitus, baseline NIHSS score, pre-treatment ASPECTS, poor collateral status, No. of passes, levels of baseline blood glucose and Hs-CRP.

**Figure 1:**
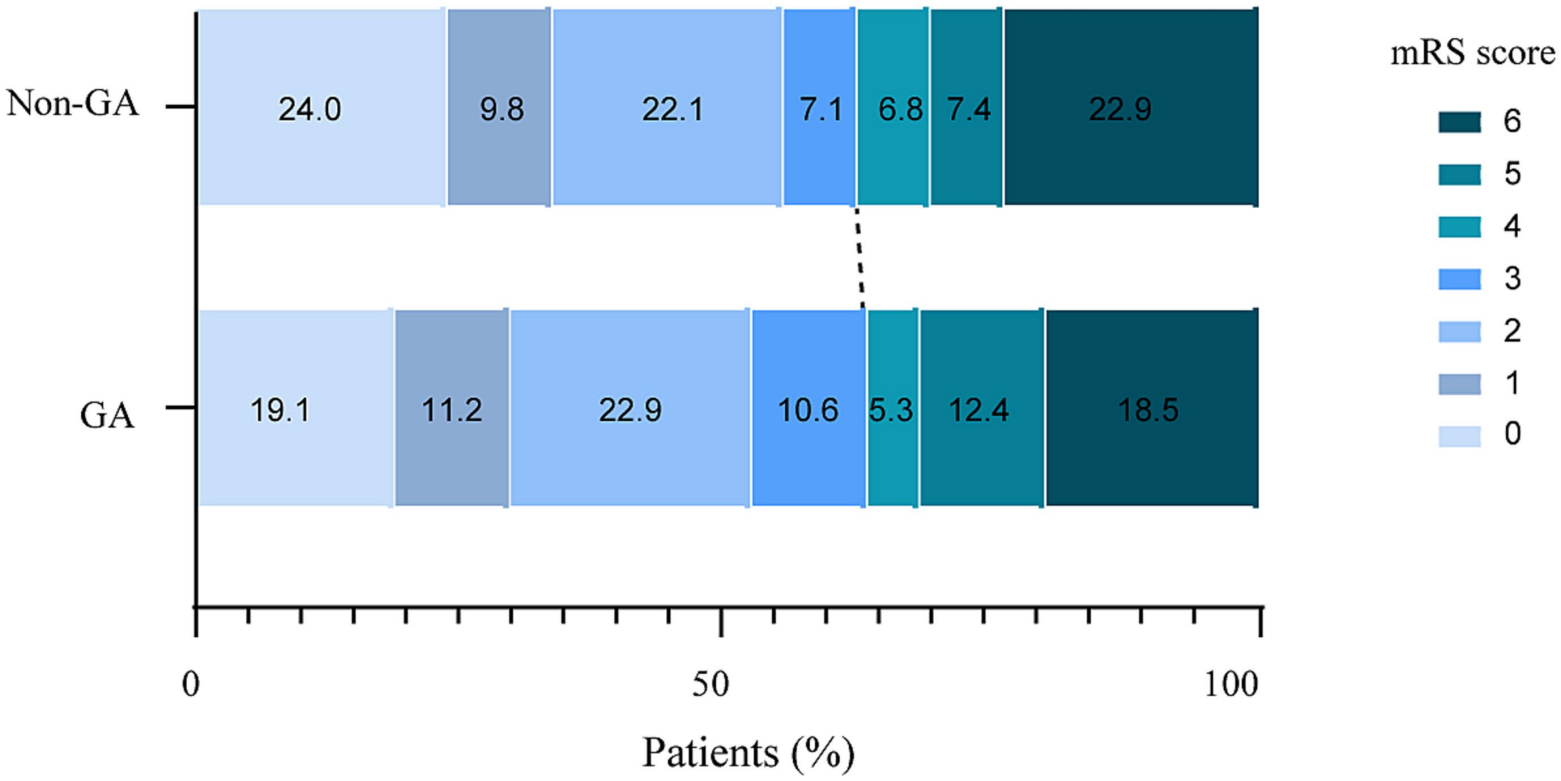
Distribution of 90-day mRS score according to the type of anesthesia. GA, general anesthesia; mRS, modified Rankin Scale.

Regarding to secondary outcomes, no significant differences were observed in distribution of mRS scores (aOR 0.932, 95% CI 0.721–1.213; *p* = 0.612; Table 3; Figure 1). No significant differences were found between the groups in the rate of successful reperfusion was not significantly different between the GA and non-GA groups (86.8% vs. 80.9%; aOR 1.372, 95% CI 0.886–2.125; *p* = 0.157), sICH (12.9% vs. 10.1%; aOR 1.444, 95% CI 0.835–2.443; *p* = 0.471), END (18.5% vs. 18.3%; aOR 0.990, 95% CI 0.658–1.490; *p* = 0.963), and mortality at 90 days (18.5% vs. 21.9%; aOR 0.758, 95% CI 0.483–1.187; *p* = 0.226). However, the risk of post-stroke pneumonia was significantly higher in the GA group compared to the non-GA group (37.9% vs. 27.5%; aOR 1.668, 95% CI 1.197–2.325; *p* = 0.003). In addition, the association of anesthesia choice with pneumonia risk was similar across subgroups stratified according to age, hypertension, diabetes, admission NIHSS score, and circulation status (Table 4).

**Table 4:** Subgroup analyses of the association between anesthesia choice and pneumonia risk.

Variable	OR (95% CI)*	*p* value
Age > 75 years	1.396 (0.840–2.320)	0.198
Age ≤ 75 years	1.784 (1.188–2.680)	0.005
With hypertension	1.869 (1.299–2.690)	0.001
Without hypertension	1.004 (0.511–1.897)	0.162
With diabetes	1.957 (1.157–3.309)	0.012
Without diabetes	1.430 (1.060–2.131)	0.049
Baseline NIHSS score > 15	1.399 (0.850–2.303)	0.186
Baseline NIHSS score ≤ 15	1.800 (1.190–2.722)	0.008
Poor circulation status	1.531 (1.002–2.399)	0.049
Good circulation status	1.723 (1.064–2.790)	0.027

CI, confidence interval; OR, add ratio.

*Adjusted for demographic characteristics, hypertension, diabetes mellitus, baseline NIHSS score, pre-treatment ASPECTS, poor collateral status, No. of passes, levels of baseline blood glucose and Hs-CRP, except for the stratified variable.

## 4 Discussion

In this multicenter, retrospective cohort study of 707 elderly stroke patients undergoing EVT, we found that the choice of anesthesia was not independently associated with a significant difference in the rate of good functional outcome at 90 days. Furthermore, we found no statistically significant differences between the GA and non-GA groups in key secondary outcomes, including successful reperfusion, symptomatic intracerebral hemorrhage, early neurological deterioration, mortality, or the overall distribution of mRS scores. However, a significant finding emerged: GA patients had a higher risk of post-stroke pneumonia than non-GA patients.

The impact of anesthesia choice on functional outcomes after thrombectomy remains a subject of debate, as evidenced by multiple randomized trials reporting divergent outcomes (5, 9–11, 24, 25). For instance, one randomized trial and a meta-analysis of 7 trials linked GA to improved functional recovery (9). Conversely, the HERMES collaborative meta-analysis (24) and a large-scale registry study demonstrated that non-GA approaches led to better clinical results (25). By focusing specifically on the elderly patients, our study adds a critical dimension to this evolving consensus. The discrepancy between earlier studies favoring GA and our null finding may be attributed to advancements in anesthetic management protocols for GA (e.g., more stringent blood pressure control) and the improved overall safety profile of EVT procedures over time. Furthermore, it is also plausible that the advanced age and significant comorbidity burden in our cohort obscured any modest treatment benefit that GA might provide in less comorbid populations. In addition, our null findings regarding sICH, mRS score distribution and mortality are consistent with a growing number of contemporary reports (5, 9–11, 26, 27). This reinforces the notion that the technical success and immediate safety of the EVT procedure itself are not predominantly determined by the choice of anesthesia in experienced centers.

It was observed in our study that GA elevated the risk of post-stroke pneumonia among elderly patients following EVT. This finding corroborates earlier studies that identified a similar association in the general population (11, 26, 27). This observation is biologically plausible and can be explained by several interconnected mechanisms. Firstly, endotracheal intubation and impaired airway protection are hallmarks of GA. The placement of an endotracheal tube can bypass natural airway defenses, potentially introducing pathogens into the lower airways (28). Moreover, neuromuscular blocking agents used in GA compromise the cough and gag reflexes-essential protective mechanisms against aspiration. Residual sedative effects after extubation can further prolong this period of vulnerability (29). Secondly, GA is known to induce immunomodulation and suppress both innate and adaptive immunity (30). Volatile anesthetics and intravenous sedatives can inhibit the function of neutrophils, macrophages, and lymphocytes, reducing the body’s ability to combat inhaled or aspirated pathogens (31). This state of immunoparalysis may be particularly detrimental in elderly stroke patients, who often experience stroke-induced immunodepression and therefore face a significantly elevated pneumonia risk. Thirdly, hemodynamic instability associated with GA may be a contributing factor. Hypotension, which is common during the induction and maintenance of GA, can cause cerebral and systemic hypoperfusion. In stroke patients, this may enlarge the ischemic penumbra and exacerbate swallowing dysfunction while also depressing the level of consciousness, thereby elevating the risk of aspiration pneumonia (32, 33). Finally, the marginally longer onset to groon puncture time observed in the GA group, though not statistically significant in our study (*p* = 0.092), aligns with known logistical challenges of administering GA (e.g., time for anesthesia team assembly and intubation). This delay could potentially prolong the duration of dysphagia and immobility, further predisposing patients to pulmonary complications. However, it is important to note that previous studies have not consistently demonstrated a significant difference in onset-to-groin puncture time between GA and non-GA groups (34, 35). The slight trend observed in our cohort may be attributable to differences in study population characteristics, institutional protocols, or the stage of the learning curve during the study period.

Several limitations of our study warrant consideration. First, its retrospective and non-randomized design introduces the potential for residual confounding, despite our comprehensive multivariate adjustments. The choice of anesthesia was at the discretion of the treating team, which may have been influenced by unmeasured patient-level factors (e.g., baseline agitation or respiratory status). Second, we lacked detailed data on specific anesthetic agents, dosages, and intraoperative hemodynamic parameters, which could influence the clinical outcomes. Third, the diagnosis of pneumonia, while based on standardized criteria, may be subject to variation in clinical practice across centers. Finally, the generalizability of our findings is confined to elderly stroke populations.

In conclusion, this multicenter study of elderly stroke patients undergoing EVT found that the type of anesthesia was not independently associated with 90-day functional outcomes, reperfusion success, hemorrhage, or mortality. However, GA significantly increased the risk of post-stroke pneumonia. Therefore, non-GA should be prioritized when feasible, particularly for patients at high risk of pulmonary complications, with the final strategy determined through individualized interdisciplinary assessment.

## Data Availability

The raw data supporting the conclusions of this article will be made available by the authors, without undue reservation.
